# Soil Moisture and Its Interaction With Temperature Determine Root Metabolomes of a Himalayan Alpine Shrub

**DOI:** 10.1111/ppl.70444

**Published:** 2025-08-12

**Authors:** Sayantika Banerjee, Nikita Rathore, Jaroslav Semerád, Tomáš Cajthaml, Zuzana Münzbergová, Dinesh Thakur

**Affiliations:** ^1^ Department of Botany, Faculty of Science Charles University Prague Czech Republic; ^2^ Institute of Botany, Czech Academy of Sciences Průhonice Czech Republic; ^3^ Institute of Microbiology of the Czech Academy of Sciences Prague 4 Czech Republic; ^4^ Institute for Environmental Studies, Faculty of Science, Charles University Prague Czech Republic

**Keywords:** climate change, eco‐metabolomics, *Rhododendron anthopogon*, untargeted metabolomic

## Abstract

Climate change profoundly impacts plants. However, our understanding of plant responses to climate largely relies on plant morphology and physiology, while plant metabolomic responses, especially those within plant roots, have received much less attention. Understanding root metabolomic variation is key to understanding cellular‐level plant responses to changing climatic conditions. In this study, we investigated the individual and interactive effects of temperature and soil moisture on the root metabolome of the alpine Himalayan dwarf shrub *Rhododendron anthopogon*. Using an untargeted metabolomics approach, we analyzed shifts in metabolomic profiles in multivariate space and identified metabolites most responsive to climatic variation. Our results revealed that soil moisture exerted the strongest influence on root metabolomic profiles, followed by the interactive effects of temperature and moisture, with temperature alone explaining the least variation. Notably, approximately 75% of metabolites significantly affected by climate responded to the interaction between temperature and moisture, suggesting that temperature effects are largely moisture‐dependent. Multiple classes of primary and secondary metabolites were influenced by climate, with flavonoids, alkaloids, and triterpenoids showing the most pronounced responses. Pathway analysis indicated the presence of several climate‐sensitive metabolites involved in key metabolic pathways. The most responsive metabolites were phenolics, glycosides, and amino acids. These metabolites formed interconnected networks, acting as hub compounds likely playing pivotal roles in regulating plant responses to climatic variability. Our findings underscore the complex interplay between climatic factors in shaping root metabolomic profiles and suggest that climate change will impact plant health and productivity, possibly also affecting plant interactions with soil biota.

## 1 Introduction

Climate change is profoundly impacting organisms globally, with plants being especially vulnerable due to their immobility (IPCC 2014). Among several approaches used in understanding plant responses to climatic stress, the functional trait‐based approach is the most popular (Henn et al. 2024; Li and Prentice 2024). However, while morphological and physiological traits have been well characterized, the biochemical traits, particularly those occurring belowground, are still underexplored. Understanding these biochemical responses is crucial, as they directly influence plant health, stress resilience, and interactions with the environment. Biochemical interactions, particularly those belowground, are essential for nutrient uptake, stress tolerance, and overall plant performance. Thus, root metabolomics, which explores the metabolic shifts in roots, reveals how plants adapt to environmental stressors at the molecular level.

Plant metabolites are broadly classified into primary (e.g., carbohydrates, proteins, and nucleic acids) and secondary metabolites (e.g., phenolics, terpenoids, and alkaloids), both crucial for growth and stress adaptation (Ober and Kaltenegger 2009; Pichersky and Gang 2000; Hartmann 2007; Wink 2018; Mishra and Pandey 2022; Sah and Sofo 2024). Primary metabolites sustain basic functions and serve as precursors for the synthesis of secondary metabolites. Under stresses such as drought, heat, or elevated CO_2_, primary metabolites such as sugars help to maintain osmotic balance and provide energy (Zandalinas et al. 2022), while secondary metabolites, such as flavonoids and anthocyanins, counter oxidative damage and absorb UV radiation (Naikoo et al. 2019). Their interplay enables efficient resource use and stress signaling, enhancing plant resilience under combined abiotic stresses (Zandalinas et al. 2022).

Most studies dealing with plant metabolomic response to climate work with the leaf metabolome. In contrast, root metabolomic responses have received less attention. Roots are essential for nutrient uptake, chemical defenses, and belowground interactions with soil organisms, which play crucial roles in sustaining plant functionality under stress (Jamieson et al. 2012; Rasmann and Agrawal 2008; Tsunoda and van Dam 2017). Root metabolomic responses to climate change can be expected to differ significantly from leaf metabolomic responses, as the soil environment is relatively stable and less variable compared to the atmosphere (Zhalnina et al. 2018; Guy et al. 2008; Badri and Vivanco 2009). Knowledge of the changes in root metabolism in response to changing environmental conditions is thus crucial for understanding belowground plant functioning. Studies using untargeted metabolomics to explore the effects of climate on root metabolome are thus needed, but we are not aware of any such study.

The majority of studies on plant metabolomic responses to climate focus on a single environmental factor, typically under controlled conditions. For instance, Baker et al. (2022) explored how temperature affects the metabolome of alpine forbs, while Guo et al. (2021) examined drought‐induced changes in maize. Dong et al. (2025) assessed heat stress responses in rice metabolomes. Though these studies provide valuable insights, they overlook the simultaneous effects of multiple climate factors in nature. Emerging evidence from leaf‐based studies indicates that temperature and moisture can interact to create non‐additive effects on plant metabolites. Wang and Wang (2023) and Georgii et al. (2017) found significant interaction effects of heat and drought stress on secondary metabolite production. In contrast, some studies reported additive effects, where temperature and moisture impact metabolites independently (Escandón et al. 2018; Sewelam et al. 2020; Secomandi et al. 2025). Whether such interactive or additive effects extend to root metabolomes, particularly in natural plant populations, remains largely unexplored, highlighting a critical knowledge gap that this study aims to address.

Elevational gradients in mountains create strong natural variation in climate over short distances, with temperature typically decreasing with increasing elevation, and precipitation either increasing or decreasing depending on regional atmospheric and topographic factors (Tito et al. 2020). These co‐varying gradients closely resemble projected climate change scenarios, making them valuable systems for studying how plants may respond to simultaneous shifts in temperature and moisture. Metabolomics has become a powerful tool for examining how environmental conditions along elevational transects influence plant cellular functioning. Such studies offer insights into the biochemical strategies plants use to adapt to changing environments, particularly through adjustments in primary and secondary metabolism (e.g., Gargallo‐Garriga et al. 2017; Henderson et al. 2023).

Although climatic gradient studies are useful to understand the role of changing temperature and moisture on plant root metabolome, the effects of climate may be confounded with the effects of soil chemistry that also change along elevational gradients. For example, studies on root exudates have shown that nutrient availability, particularly nitrogen and phosphorus, significantly influence the exudation of amino acids and carbohydrates from roots, affecting nutrient mobilization, plant adaptation, and plant‐microbe interactions (Carvalhais et al. 2013; Brown et al. 2022; Kangi 2024). Soil chemical properties, including pH and nutrient content, have also been shown to have effects on plant enzyme activities and microbial biodiversity, which, in turn, impact root metabolomic profiles (Cheng et al. 2018; Delory et al. 2023; Zhao et al. 2019). Whether the effects of soil chemistry interfere with the effects of climate on plant root metabolomic profiles, however, remains to be explored.

To address how climatic factors affect plant root metabolome, this study investigates the effects of temperature and moisture, and their interaction on root metabolites in alpine Himalayan dwarf shrub *Rhododendron anthopogon* (Ericaceae). This species, native to high‐altitude environments, provides an ideal model for examining root metabolomic responses along natural climatic gradients. This research attempts to clarify the metabolomic profiles that allow plants to fight climatic stress. Our study also controls for possible confounding effects of soil chemistry occurring along the climatic gradients.

Specifically, we aim to answer the following questions.

(Q1) How do root metabolomic profiles vary along natural climatic gradients in a high‐elevation alpine dwarf shrub‐line species, and do temperature and moisture individually or interactively affect these profiles? (Q2) What specific metabolites and associated metabolomic processes are affected by climatic factors and how? (Q3) How does soil chemistry affect root metabolomic profiles, and does controlling for soil chemistry affect the conclusion on the effects of climate?

We hypothesize that H1: Root metabolomic profiles are driven by both individual and interactive effects of temperature and soil water availability. Given that low temperature is a primary climatic filter limiting plant growth in alpine areas, we expect temperature to have a stronger effect, but also interact with moisture. H2: Climatic factors, through their interactive effects, induce directional shifts in specific metabolites related to abiotic and biotic stress responses. For example, under conditions of decreased temperature and moisture, metabolites playing a role in abiotic stress tolerance, such as amino acids, are expected to increase. Conversely, defense‐related metabolites, including glycosides and flavonoids, are anticipated to rise under warmer temperatures and higher water availability. H3: Soil chemistry significantly influences root metabolomic profiles, and a substantial portion of the variation in root metabolome is explained by climatic factors, which are mediated by soil chemistry.

## 2 Materials and Methods

### 2.1 Study Species

*Rhododendron anthopogon* (D. Don) is an evergreen, broad‐leaved shrub belonging to the family Ericaceae, and is a dominant component of high‐altitude vegetation across the Western Himalayas (Rathore et al. 2021; Basnett and Ganesan 2022; Thakur et al. 2024). It typically forms dense patches along the alpine shrubline, often occupying open, sunny slopes between 3000 and 5000 m (Rathore et al. 2022). Ecologically, *R. anthopogon* plays a key role in stabilizing alpine soils and contributes to nutrient cycling by forming ericoid mycorrhizal associations that enhance nutrient acquisition under cold, nutrient‐poor conditions (Kohout 2017).

The species follows a slow‐growth, conservative resource‐use strategy, making it well adapted to environments characterized by extreme temperature fluctuations, high UV radiation, and seasonal drought, hallmarks of Himalayan alpine habitats (Körner and Kèorner 1999). In addition to its ecological role, *R. anthopogon* holds significant ethnobotanical value. Its leaves are traditionally used in Tibetan and Himalayan medicine for treating respiratory issues, fever, and inflammation, owing to its rich profile of essential oils and secondary metabolites (Kala 2005). These features, broad ecological amplitude, cultural relevance, and demonstrated biochemical resilience, make *R. anthopogon* an ideal model species for examining how alpine plants adapt at the metabolomic level to climatic variability.

### 2.2 Study Area

**FIGURE 1:**
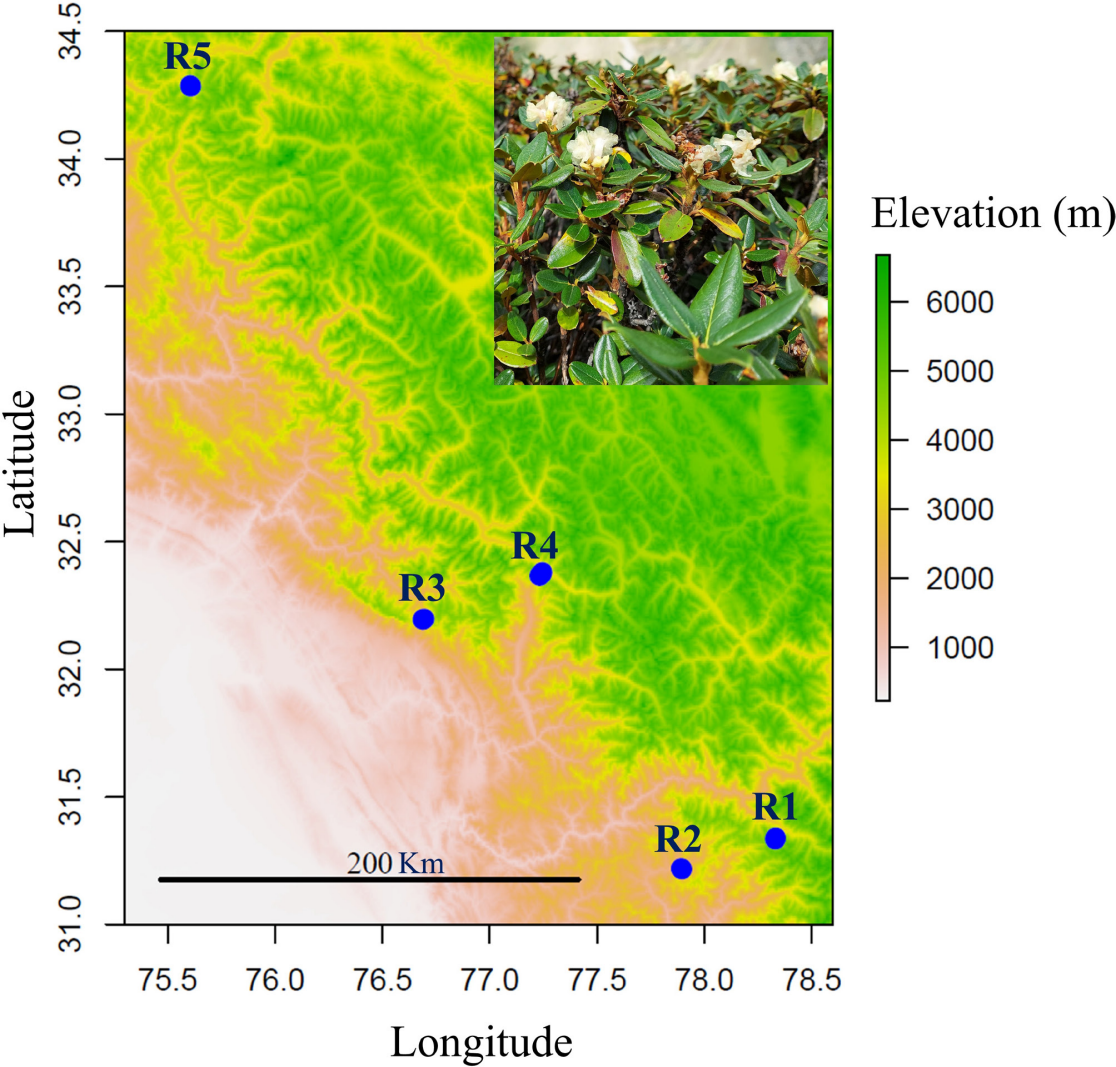
Map showing the whole study area with five regions (R1–R5). Photo in the upper right shows *Rhododendron anthopogon*, the focal species. In each region, three populations along an elevation gradient were studied.

The study was conducted in the Western Himalayan region of India. Five geographically dispersed elevational transects with varying mean annual precipitation values were chosen (Table 1). High mountain peaks and valleys served as natural barriers that separated the five studied regions in this study, which had aerial distances varying from 60 to 400 km (Figure 1). At each transect, populations from three locations along an elevation gradient (high, middle, and low) were selected, resulting in 15 studied populations (see Table 1 for details). These ranges in soil water and temperature provided us with appropriate settings to investigate individual and joint effects of soil water availability and temperature on plants.

**TABLE 1:** Growing season temperature (°C) and moisture (volumetric) of each population.

Site	Elevation (m.a.s.l.)		Temperature (°C)	Moisture (v/v)
R1	High	4200	8.43	0.26
	Middle	3900	10.74	0.23
	Low	3600	11.18	0.23
R2	High	3900	7.66	0.30
	Middle	3750	10.56	0.23
	Low	3600	12.24	0.23
R3	High	4000	9.64	0.36
	Middle	3800	10.07	0.35
	Low	3600	10.97	0.38
R4	High	4050	9.56	0.30
	Middle	3600	10.14	0.30
	Low	3250	11.13	0.32
R5	High	3950	7.42	0.31
	Middle	3800	8.08	0.18
	Low	3600	9.53	0.23

### 2.3 Root Sampling

The sampling for the study was conducted from August to September in 2021 (between the 8th of August and the 3rd of September). At each population, three plots covering an area of approximately 100 m^2^ were selected from a vegetation patch dominated by *R. anthopogon*. Within each plot, three root samples were taken from mature, fully grown, and visually healthy individuals to ensure consistency in developmental stage and physiological condition. Selected individuals were uniform in size, lacked dead branches, and showed no signs of herbivory, disease, or wilting. This resulted in nine samples per population. The sampled plant individuals within a plot were spaced at least 5 m apart from each other, and the plots themselves were at least 20 m apart. A total of 135 (5 population × 3 elevations × 3 plots × 3 samples) fine root samples were collected. Fine roots were separated from woody main roots. Fine roots are typically defined as the smaller, distal portions of the root system, usually less than 2 mm in diameter. These roots are short‐lived, highly dynamic, and play a primary role in nutrient and water uptake, root exudation, and interactions with soil microbes (Pregitzer et al. 2002; McCormack et al. 2015). In contrast, the main or coarse roots serve primarily structural and transport functions and exhibit relatively low metabolic activity. The fine roots were cleaned with tap water and stored in paper bags with silica gel. Later, the dried roots were ground into a fine powder using a grinding mill.

### 2.4 Metabolite Profiling

#### 2.4.1 Methanolic Extraction

Root metabolite extraction followed Marr et al. (2021) with minor modifications. For each sample, 500 μL of methanol: water (80:20 v/v, HPLC‐grade, Honeywell) was added to 20 mg of finely ground, dried root tissue in 2 mL tubes. Samples were vortexed (2 × 2 min), incubated at −20°C for 24 h, then vortexed again and centrifuged (15 min at 15,000 *g* (rcf)). The supernatant was collected and the extraction repeated; pooled supernatants were combined. A 320 μL aliquot was mixed with 80 μL of water:formic acid (99.9:0.1 v/v, Sigma‐Aldrich) and stored at −20°C for ≥ 48 h. Three blanks (without tissue) and one quality control (QC) sample—prepared by pooling 20 μL from each sample—were processed similarly to monitor contamination and instrument variability.

#### 2.4.2 LC–MS Analysis

A mass spectrometer and liquid chromatography system (6546 LC/Q‐TOF, Agilent) were used to acquire the data. Five‐microliter aliquots of analytical samples were separated at 40°C using a binary gradient elution at a flow rate of 0.4 mL/min on an Acclaim Rapid Separation Liquid Chromatography (RSLC) 120 column (150 × 2.1 mm, particle size 2.2 μm, Thermo Fisher Scientific). Solvent A consisted of water and formic acid (99.9:0.1, v/v), while Solvent B consisted of acetonitrile and formic acid (99.9:0.1, v/v; acetonitrile: Merck). The elution gradient was as follows: Initial conditions of 95% Solvent A were held for 1 min, then decreased to 80% A over the next 5 min (up to minute 6) and held for 1 min; at minute 7, Solvent A was further decreased to 75% over 2 min, then to 5% over the next 8 min, and held for 2 min (up to minute 19). Finally, Solvent A was reduced to 0% in 1 min and held for 5 min to elute non‐polar compounds. The total run time was 25 min. After the gradient elution, the column was re‐equilibrated to the starting condition over 4 min. Separated molecular features were collected in both positive and negative ionization modes.

#### 2.4.3 Spectra Processing and Feature Extraction

The MetaboanalystR (Pang et al. 2024) and OptiLCMS (Pang et al. 2022) packages in R were employed to process the data. MS spectral data was converted to mzXML in centroid mode using Proteowizard (Pang et al. 2024). Processing included peak picking, alignment, and gap filling to generate MS feature tables. We detected a total of 11,218 metabolomic features in positive ionization mode and 9823 in negative ionization mode. Features were filtered using Metaboanalyst 6.0 tools (https://www.metaboanalyst.ca/), excluding those present in blank samples, absent in over 80% of samples, or showing more than 25% relative standard deviation in QC samples. This resulted in a refined dataset with 4759 features in positive and 588 in negative ionization mode. Missing values were addressed by filling empty cells with 1/5 of the minimum value for each metabolite. QC samples were adjusted for systematic differences in LC–MS analysis prior to statistical evaluations.

### 2.5 Climate Data Recording and Analysis

In our study system, the climatic parameters for each population were recorded using 2 TMS4 dataloggers (Wild et al. 2019) for every 15 min, each day over a period of 1 year. It included air temperature, surface temperature, soil temperature, and soil moisture. The daily average, maximum, and minimum of soil, surface, and air temperature were calculated, and then the annual average temperature of each variable was calculated for each population. Apart from this, we also extracted the daily average, maximum, and minimum temperatures of each climatic parameter during the growing season from the month of May to September. The number of degree days for each population was also calculated following the averaging method by McMaster and Wilhelm (1997) and Nugent (2005). For our study, we used 5°C as the base temperature and subtracted that value from the daily average soil temperature. After this, we counted the number of days with more than 5°C average temperatures to calculate the number of growing degree days. The moisture‐related parameters included the annual average moisture and average moisture during the growing season (May to September). We checked the correlation between all climatic variables using the corrplot package (Wei et al. 2017) in R, by making a correlation matrix (Figure S1). Based on correlation strength, we retained only one variable from all showing a correlation coefficient above 0.7. We retained growing season temperature and growing season‐soil water content to represent the temperature and moisture in the analyses and thus refer to these variables as temperature and moisture in subsequent text.

### 2.6 Soil Chemical Analysis

We collected three (one per plot) soil samples (each ~500 g) per population. These samples were collected around the same individuals as used for root and rhizosphere analysis, but samples from all three individuals per plot were combined to get one sample per plot. These soil samples were air‐dried and stored in paper bags for further analysis. For analysis, the air‐dried soil samples were sieved through a mesh size of 2 mm, and then pH, electrical conductivity (EC), total carbon, and nitrogen, and available phosphorus were measured following methods described in Carter and Gregorich (2007). K content was measured using flame photometry (Carter and Gregorich 2007).

### 2.7 Statistical Analyses

The statistical analysis of the data was performed using R (version 4.2.0). All analyses were done separately for positive and negative ionization mode datasets.

#### 2.7.1 Quantification of Root Metabolite Diversity

We quantified metabolite diversity from positive‐ and negative‐mode LC–MS data using four indices: Richness, Shannon, functional specialization (calculated as the distance of each sample to the multivariate trait centroid), and functional β‐dispersion (the distance to the sample's plot centroid, computed using the betadisper() function in the vegan package) (Oksanen et al. 2007). We initially also included Evenness but excluded it from further analyses due to strong collinearity with Shannon (Wei et al. 2017). For each ionization mode, we merged the diversity indices with environmental metadata and fitted linear mixed‐effects models using the lme4 package (Bates et al. 2015), treating Plot as a random intercept. We fitted two models per index: a climate model including temperature, moisture, and their interaction, and a full model that additionally included six soil predictors (pH, P, N, EC, K, and total C). We assessed the significance of fixed effects using Type III ANOVA with the Satterthwaite approximation, implemented via the lmerTest package (Kuznetsova et al. 2017).

#### 2.7.2 Determinants of Metabolomic Composition

We performed Redundancy Analysis (RDA) to determine the effects of climatic factors on the root metabolome, using temperature and moisture, and their interaction as predictors. Climatic effects were tested both with and without soil chemistry parameters (soil P, N, K, C, EC, and pH) as additional predictors. The significance of the effects was assessed using a permutation test, where all nine samples from the three plots per population were kept together, and the populations were freely permuted among each other. We also ran alternate RDA models using sampling time as a covariate. However, the patterns of climatic effects remained unchanged in these models. Therefore, all results are based on models without controlling for sampling time. All these analyses, including RDA, were conducted in R using the “vegan” package (Oksanen et al. 2007).

#### 2.7.3 Relationship of Different Metabolites With Climate

We used mixed‐effect models to investigate the effect of climatic factors (temperature, moisture, and their interaction) on single metabolites. While fitting the models, we used soil chemistry as a covariate and plot as a random factor. The significant metabolites were determined by applying the False Discovery Rate (FDR) correction (using the Benjamini‐Hochberg method) to account for multiple hypothesis testing. We set the FDR threshold at 0.1 (Ayele and Zewotir 2016).

#### 2.7.4 Annotation of Metabolites Significantly Affected by Climate

We annotated the metabolites significantly affected by climate. For this, data‐dependent LC–MS/MS analysis was performed on a pooled sample (used as a quality control in LC–MS analysis) to collect the necessary data. Subsequently, we used MS‐DIAL (Tsugawa et al. 2015) software to putatively annotate the metabolites detected in LC–MS/MS mode data. LC–MS/MS data was processed by aligning retention times, detecting peaks, and deconvoluting spectra. The annotation was then done by matching detected compounds against freely available libraries at https://systemsomicslab.github.io/compms/msdial/main.html. A mass accuracy threshold of 10 ppm was used in the annotation process. After annotation, the metabolites were assigned to their chemical classes.

#### 2.7.5 Metabolomic Pathway Analysis of Metabolites Significantly Affected by Climate

Metabolomic pathway analysis was conducted using MetaboAnalyst 6.0. The input dataset included metabolite names and identifiers (HMDB, KEGG, PubChem), formatted as a compound concentration table. A total of 161 metabolites were used in the later processes. Among these, 23 pathways involving 20 metabolites were finally used.

#### 2.7.6 Correlation Network Analysis

A correlation analysis was conducted using Pearson correlation coefficients, computed with the rcorr function from the *Hmisc* package (Harrell Jr and Harrell Jr 2019). Corresponding *p*‐values were extracted, and multiple testing correction was applied using the False Discovery Rate (FDR) method to control for Type I errors. A correlation‐based network was then constructed from the filtered correlation matrix, retaining only significant correlations (adjusted *p* < 0.05). The *qgraph* package (Epskamp et al. 2012) was used to generate an undirected, weighted adjacency matrix. Node colors were assigned according to metabolite classes, while node sizes were scaled based on node degree (number of connections). The network graph was visualized using the *qgraph* package (Epskamp et al. 2012). Key network parameters, including modularity and edge density, were computed to assess the structural properties of the network.

## 3 Results

### 3.1 Effect of Climatic Factors on Metabolome Diversity

In both ionization modes, climate variables alone did not significantly explain variation in any diversity index, except for β‐dispersion in the positive mode, which showed significant effects of temperature, moisture, and their interaction (Table 2). Specifically, β‐dispersion decreased with increasing temperature at low moisture and increased with temperature at high moisture levels (Figure S2a). When soil variables were included as additional predictors, more significant patterns emerged across both ionization modes. In the positive mode, richness was also negatively associated with soil electrical conductivity (EC). At the same time, functional specialization decreased with higher soil carbon and with temperature (Table 2; Figure S2b–d). In the negative mode, Shannon diversity increased with soil EC (Figure S2e), and specialization increased with higher soil nitrogen (Figure S2f). β‐Dispersion in the negative mode decreased with increasing soil EC and temperature (Figure S2g,h). Richness remained unresponsive to either climatic or edaphic factors in both modes (Table 2).

**TABLE 2:** Results of diversity analysis testing the effect of temperature (T), moisture (M), and their interaction alone and together with six soil predictors on the root metabolome diversity.

Diversity index		Richness				Shannon				Specialization				Beta‐dispersion			
Ionization mode		Positive		Negative		Positive		Negative		Positive		Negative		Positive		Negative	
		*F*	*p*	*F*	*p*	*F*	*p*	*F*	*p*	*F*	*p*	*F*	*p*	*F*	*p*	*F*	*p*
Clim. var.	T	0.31	0.58	0.3	0.58	0.61	0.44	0.29	0.59	0.04	0.84	0.14	0.71	**6.4**	**0.01**	2.91	0.1
	M	0.36	0.55	0.06	0.81	0.58	0.45	0.48	0.49	0.04	0.84	0.09	0.76	**4.45**	**0.04**	2.36	0.13
	T × M	0.25	0.62	0.29	0.59	0.56	0.46	0.36	0.55	0.01	0.92	0.26	0.61	**5.74**	**0.02**	3.42	0.07
Clim. + soil var.	pH	0.26	0.61	0.10	0.76	0.77	0.39	0.07	0.79	0.63	0.43	0.01	0.90	0.05	0.83	0.66	0.42
	P	2.53	0.12	0.01	0.93	1.80	0.19	1.22	0.27	1.63	0.21	0.14	0.71	0.99	0.32	3.43	0.07
	N	0.03	0.86	0.91	0.35	0.01	0.91	0.29	0.59	0.46	0.50	**4.26**	**0.05**	1.92	0.17	2.09	0.16
	EC	**4.13**	**0.05**	1.22	0.28	1.29	0.26	**4.89**	**0.03**	1.45	0.24	0.16	0.69	0.96	0.33	**4.51**	**0.04**
	K	2.00	0.17	0.35	0.56	0.40	0.53	2.75	0.10	0.99	0.33	1.13	0.29	0.03	0.86	1.44	0.24
	C	0.49	0.49	2.22	0.15	0.01	0.93	0.02	0.89	**8.30**	**0.01**	0.12	0.73	1.10	0.30	0.39	0.53
	T	1.09	0.30	0.03	0.86	0.67	0.42	3.22	0.08	**5.89**	**0.02**	1.91	0.18	3.18	0.08	**4.60**	**0.04**
	M	0.88	0.35	0.08	0.79	0.92	0.34	3.32	0.07	3.16	0.08	2.07	0.16	2.60	0.11	3.86	0.06
	T × M	1.07	0.31	0.07	0.80	0.76	0.39	3.51	0.06	3.85	0.06	1.91	0.18	2.94	0.09	**4.91**	**0.03**

*Note:* Bold values represent the significant *F* and *P* values.

### 3.2 Effect of Climatic Factors on Metabolome Differentiation

**FIGURE 2:**
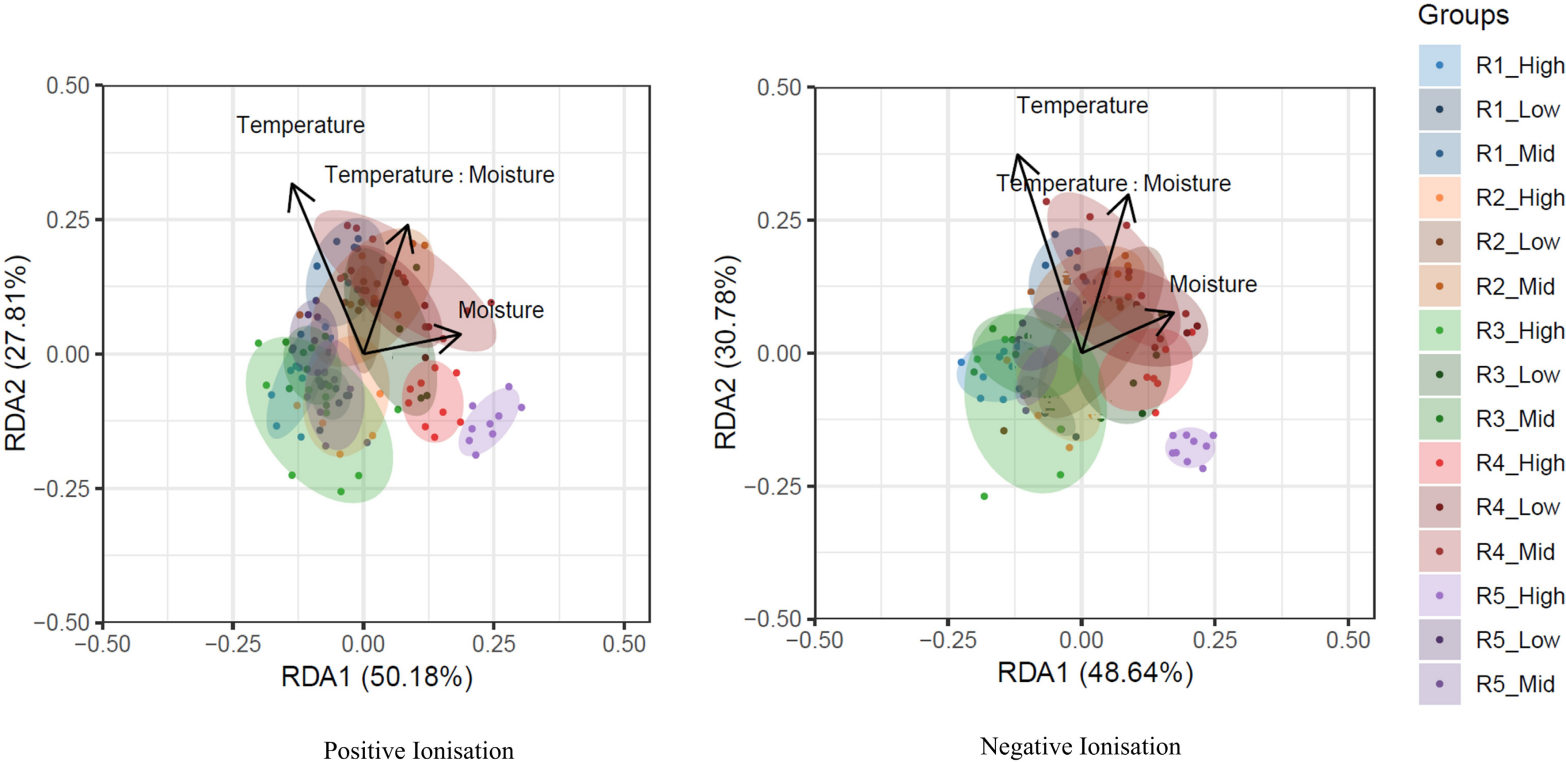
RDA plots showing the effect of temperature (T), moisture (M), and their interaction on root metabolome (with soil chemistry as covariates). The reported percentage (%) variation along the first and second axes represents the percentage out of the total proportion explained in RDA. Shaded areas depict the 95% confidence intervals of the trait space occupied by each population. The color groups assigned to each population are based on geographical regions of occurrence of populations (R1–R5), with three elevations.

In the RDA model with only climate as predictors, only moisture had a significant effect in both the ionization modes (Table 3). When soil chemical parameters (soil pH, N, P, K, EC, and C) were taken as additional predictors, the effect of moisture remained significant and explained the most variation. This model also showed that the interaction between climatic factors (temperature × moisture) had a significant effect on the root metabolome (Table 3; Figure 2). Temperature alone did not have a significant effect on the root metabolome. Among soil variables, only soil EC showed significant effects in both ionization modes (Table 3).

**TABLE 3:** Results of redundancy analysis testing the effect of temperature (T), moisture (M), and their interaction alone and together with six soil predictors on the root metabolome.

Ionization mode		Positive			Negative		
		*F*	*p*	% var	*F*	*p*	% var
Clim. var.	T	4.40	0.12	—	5.64	0.18	—
	M	**5.18**	**0.04**	**3.6**	**8.8**	**0.01**	**6**
	T × M	2.23	0.94	—	2.28	0.98	—
Clim. + soil var	pH	3.41	0.24	—	5.1	0.12	—
	P	4.27	0.09	—	4.73	0.24	—
	N	3.61	0.26	—	6.78	0.052	—
	EC	**4.65**	**0.03**	**3.7**	**7.99**	**0.008**	**5**
	K	3.13	0.37	—	4.65	0.19	—
	C	3.17	0.38	—	4.09	0.34	—
	T	3.25	0.12	—	3.87	0.09	—
	M	**4.48**	**0.01**	**4.48**	**4.34**	**0.03**	**3.2**
	T × M	**3.54**	**0.03**	**3.54**	**3.99**	**0.04**	**2.9**

*Note:* Bold values represent the significant *F* and *P* values.

### 3.3 Effect of Climatic Factors on Specific Metabolites

In the positive ionization mode dataset, a total of 568 metabolites were significantly influenced by temperature, 281 by moisture, and 496 by their interaction (Figure S3a). In the negative ionization mode dataset, 78 metabolites were affected by temperature, 44 by moisture, and 73 by their interaction (Figure S3b). Interestingly, while a substantial number of metabolites were significantly impacted by these climatic factors, the majority were not uniquely affected by a single factor. Instead, the same metabolites were influenced by both climatic variables and their interaction (Figure S3a,b). This indicates a complex interplay between temperature and moisture in regulating metabolomic processes. Out of all the metabolites affected by climate, around 75% have been affected by the interaction between moisture and temperature in both ionization modes.

**FIGURE 3:**
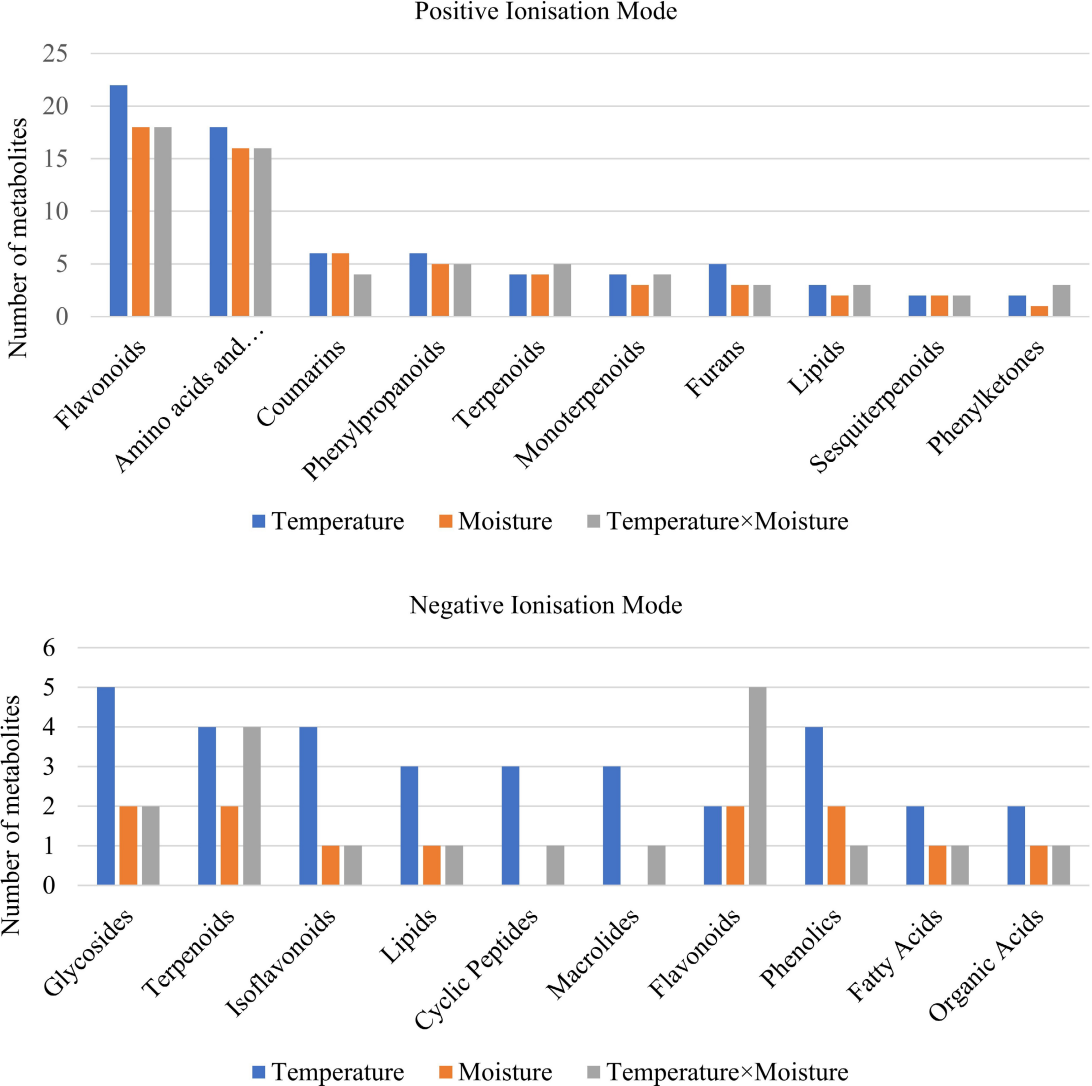
Bar plot showing the number of metabolites from each metabolite class affected by temperature, moisture, and their interaction (temperature × moisture) for both ionization mode datasets: Positive and negative.

Among the metabolites significantly influenced by climate in both modes, almost 30% were putatively identified. These annotated metabolites, which were significantly affected, belonged to both primary and secondary metabolite classes (Figure 3). Among the metabolite classes, flavonoids were the most affected, followed by coumarins, phenylpropanoids, and terpenoids for the positive ionization mode dataset (Figure 3). In the negative ionization mode dataset, the metabolites most affected were glycosides, followed by flavonoids and terpenoids (Figure 3). The detailed list of metabolite classes annotated for both ionization modes has been provided in the Supporting Information (Tables S1 and S2).

### 3.4 Metabolomic Pathway Affected by Climate

The metabolomic pathway analysis revealed that the metabolites significantly affected by climate and annotated were associated with 23 metabolomic pathways (Table 4; Figure S4). The most strongly climate‐impacted metabolomic pathways (based on bubble color, where red indicates highly significant pathways and yellow represents less significant ones) were valine, leucine, and isoleucine degradation and synthesis (bubble no. 1 and 3), followed by monoterpenoid biosynthesis (bubble no. 4), glycerophospholipid metabolism (bubble no. 5), phenylpropanoid biosynthesis (bubble no. 6), flavonoid biosynthesis (bubble no. 8), and vitamin B6 metabolism (bubble no. 9). The strongest pathways (bubble size) were pantothenate and CoA biosynthesis (bubble no. 19), followed by arginine biosynthesis (bubble no. 13), glycosylphosphatidylinositol (GPI)‐anchor biosynthesis (bubble no. 20), and cyanoamino acid metabolism (bubble no. 21) (Table 4; Figure S4). The metabolite names from each of the pathways are presented in Table 4.

**TABLE 4:** Metabolomic pathways, the specific metabolites related to particular pathways and the number of metabolites (hits) that were affected by climate from each pathway in this study.

Bubble no.	Pathway name	Metabolite name	Hits
1	Valine, leucine, and isoleucine degradation	Isoleucine; leucine, methylmalonic acid	3
2	Alanine, aspartate, and glutamate metabolism	l‐Alanine; l‐asparagine	2
3	Valine, leucine, and isoleucine biosynthesis	Isoleucine; leucine	2
5	Glycerophospholipid metabolism	Phosphatidylethanolamine	2
6	Phenylpropanoid biosynthesis	Ferulic acid; syringin	2
8	Flavonoid biosynthesis	Myricetin; taxifolin	2
18	Purine metabolism	Adenosine; guanosine	2
4	Monoterpenoid biosynthesis	Beta‐myrcene	1
7	Tropane, piperidine, and pyridine alkaloid biosynthesis	Tropine	1
9	Vitamin B6 metabolism	Pyridoxine	1
10	Selenocompound metabolism	l‐Alanine	1
11	Butanoate metabolism	Butyric acid	1
12	Beta‐alanine metabolism	Pantothenic acid	1
13	Arginine biosynthesis	Ornithine	1
15	Propanoate metabolism	Propionic acid	1
16	One carbon pool by folate	Adenosine	1
17	Carbon fixation by Calvin cycle	l‐Alanine	1
19	Pantothenate and CoA biosynthesis	Pantothenic acid	1
20	Glycosylphosphatidylinositol (GPI)‐anchor biosynthesis	Phosphatidylethanolamine	1
21	Cyanoamino acid metabolism	l‐Asparagine	1
22	Arginine and proline metabolism	Ornithine	1
23	Porphyrin metabolism	Protoporphyrin IX	1

### 3.5 Direction of Changes in Specific Metabolites in Response to Climatic Factors

#### 3.5.1 Metabolites Involved in Metabolomic Pathways (in Table 4)

**FIGURE 4:**
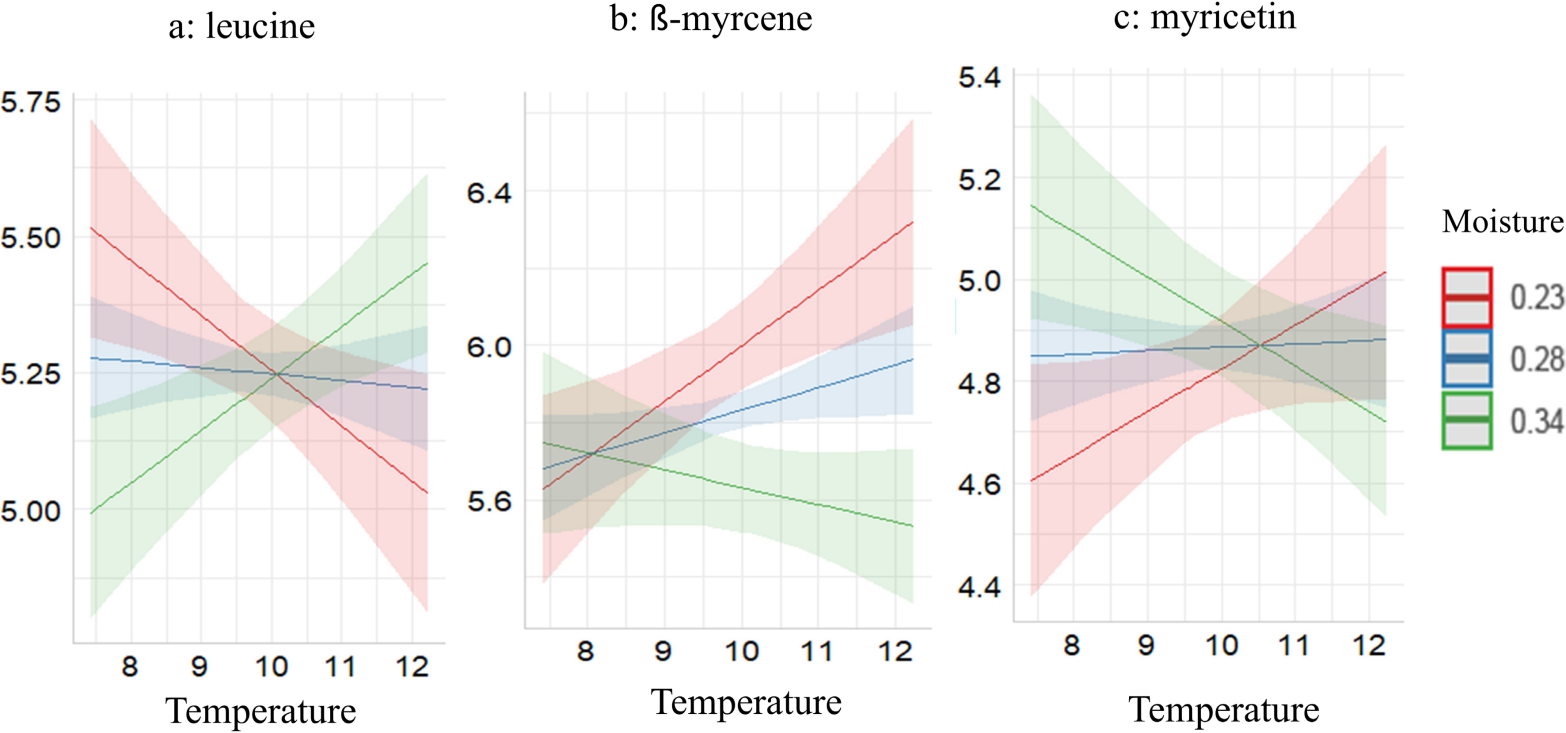
Patterns of changes of leucine (a), ß‐myrcene (b), and myricetin (c) in response to temperature, moisture, and their interaction in some of the metabolites present in the metabolomic pathway analysis. The plots are based on the significant effects detected in the linear mixed effects models.

Among the metabolites significantly affected by climate from the pathways, leucine (Figure 4a), isoleucine (Figure S5a), ferulic acid (Figure S5b), syringin (Figure S5c), and pantothenic acid (Figure S5d) increased with increasing temperature but only under high soil moisture content, while the opposite trend was observed under low moisture content. On the other hand, the metabolites that showed an increasing trend with an increase in temperature at low soil moisture content and the opposite under high soil moisture content include beta‐Myrcene (Figure 4b), myricetin (Figure 4c) phosphatidylethanolamine (Figure S5e), taxifolin (Figure S5f), pyridoxine (Figure S5g), asparagine (Figure S5h), and ornithine (Figure S5i).

#### 3.5.2 Other Metabolites

**FIGURE 5:**
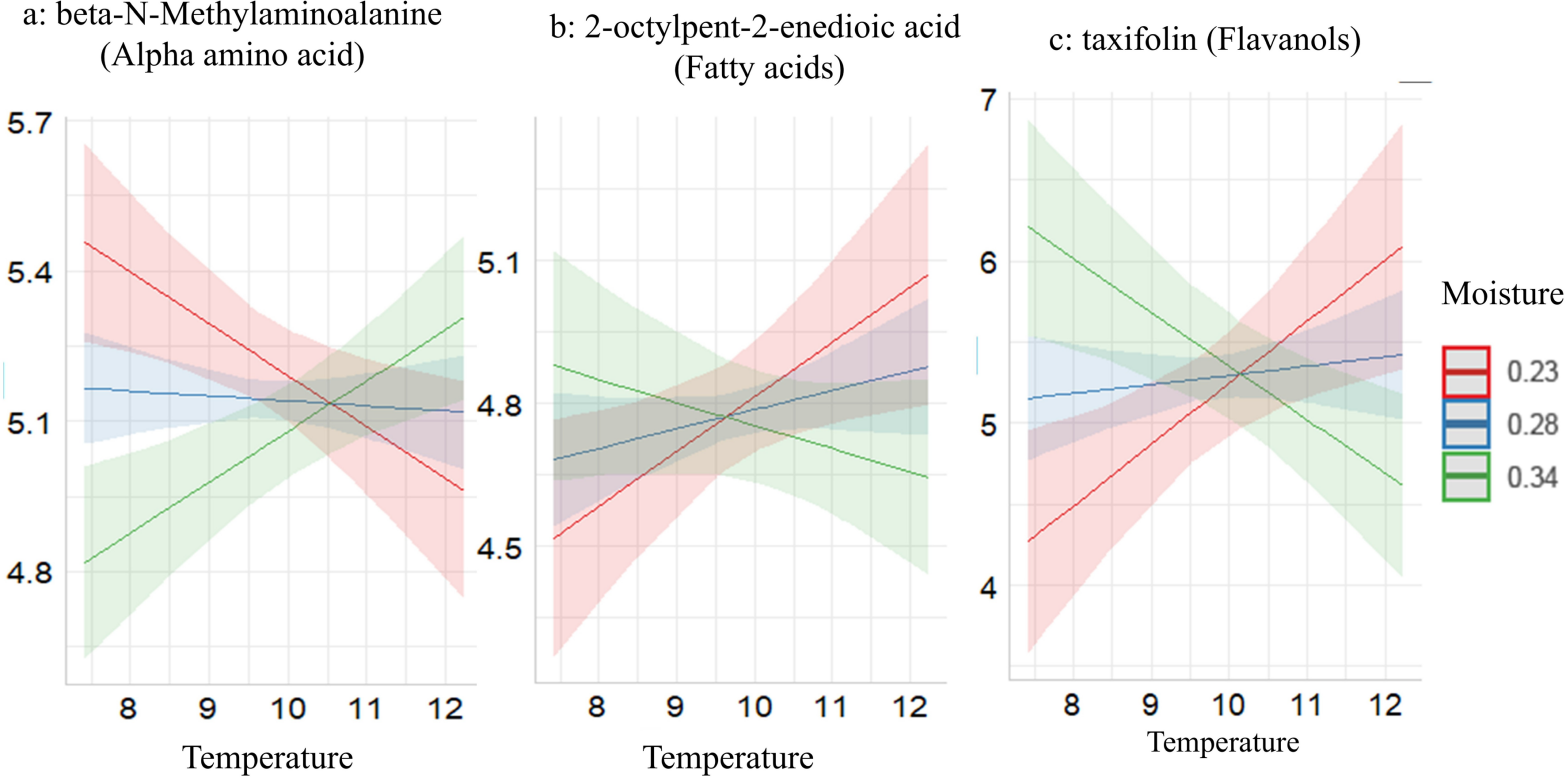
Patterns of changes of beta‐N‐methylaminoalanine (alpha amino acid) (a), 2‐octylpent‐2‐enedioic acid (fatty acids) (b), and taxifolin (flavanols) (c) in response to temperature, moisture, and interaction in some of the detected and annotated metabolites. The plots are based on the significant effects detected in the linear mixed effects models.

Among the many other metabolites that were not represented in pathways above, primary metabolites, including fatty acids (Figure 5b) and arginine & derivatives (Figure S6a), exhibited an increasing trend with rising temperatures and low moisture levels. In contrast, alpha amino acids (Figure 5a), and saccharolipids (Figure S6b) demonstrated an opposite trend. Secondary metabolites, such as flavanols (Figure 5c), phenolic glycosides (Figure S6d), and coumarin glycosides (Figure S6e), showed a decreasing pattern with elevated temperatures and high moisture levels. In contrast, triterpenoids showed an increasing pattern with elevated temperatures at higher moisture levels (Figure S6c).

#### 3.5.3 Correlation Network Among the Metabolites

The correlation network analysis conducted in both positive and negative modes (Figure S7a,b) revealed a complex web of interactions among metabolites, highlighting the intricate nature of plant metabolomic networks. Relationships spanned within and between various metabolite classes without any specific clustering of specific metabolite classes. Several hub metabolites (those with a higher number of connections) were also revealed. Their high connectivity suggests that they may be key regulators in response to climatic factors, ensuring optimal functioning of plants in diverse climates.

**FIGURE 6:**
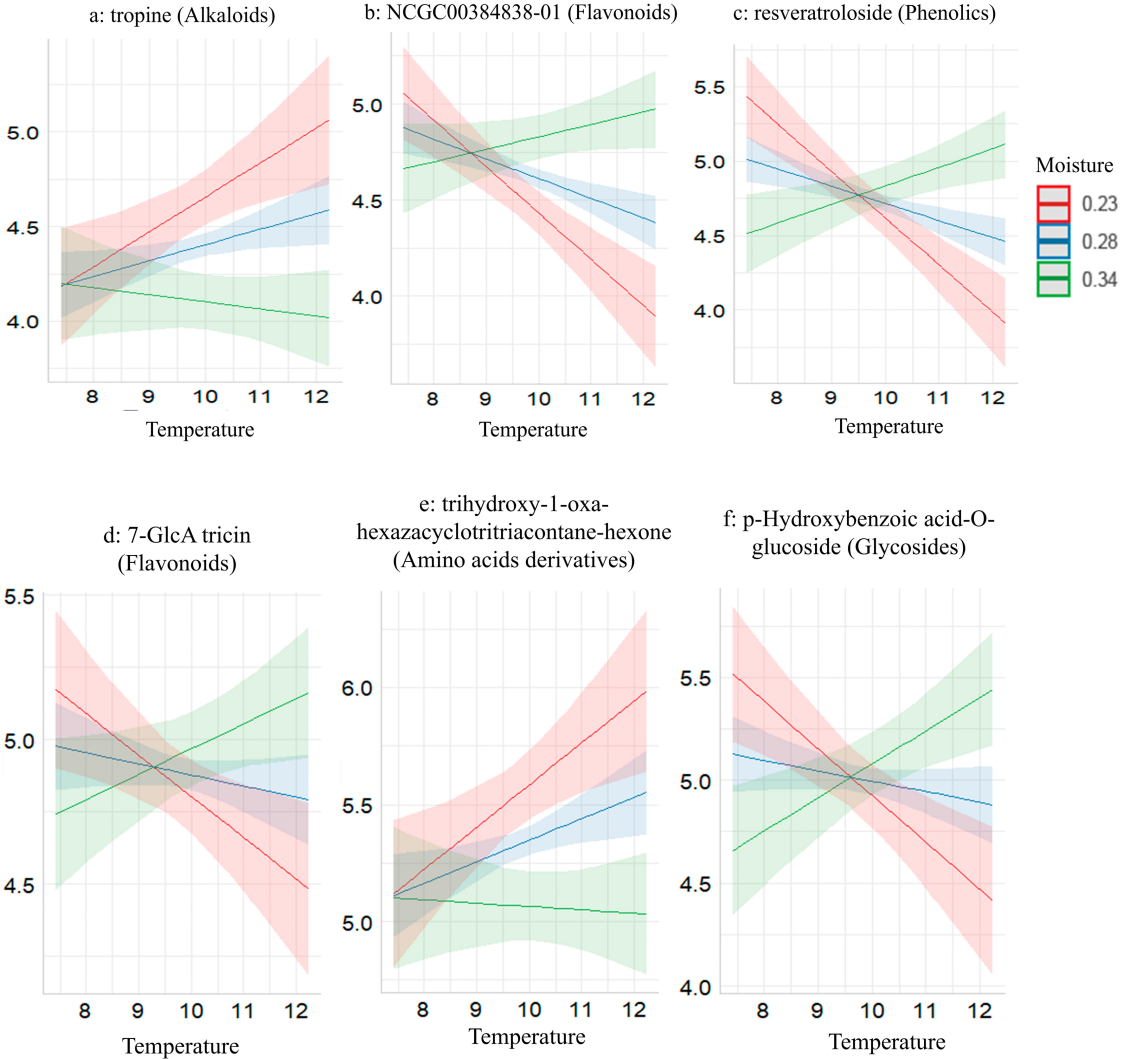
Patterns of changes of tropine (alkaloids) (a), NCGC00384838‐01 (flavonoids) (b), resveratroloside (phenolics) (c), 7‐GlcA tricin (flavonoids) (d), trihydroxy‐1‐oxa‐hexazacyclotritriacontane‐hexone (amino acids derivatives) (e), and p‐hydroxybenzoic acid‐O‐glucoside (glycosides) (f) in response to temperature, moisture, and interaction in some of the hub metabolites for both modes given in Figure S7a,b. The plots are based on the significant effects detected in the linear mixed effects models.

In the positive ionization mode, the key hub metabolites include resveratroloside (node 95, class phenolics), NCGC00384838‐01 (node 27, class flavonoids), and tropine (node 1, class alkaloids). In the negative ionization mode, the metabolites are 7‐GlcA tricin (node 1, class flavonoids), trihydroxy‐1‐oxa‐hexazacyclotritriacontane‐hexone (node 29, class aminoacids derivatives), and *p*‐Hydroxybenzoic acid‐O‐glucoside (node 12, class glycosides). Direction of change in response to climatic factors in key hub metabolites is presented in Figure 6a–f.

## 4 Discussion

This study provides novel insights into root metabolomic responses to natural climatic gradients, using the high‐altitude evergreen shrub *R. anthopogon* as a model. We demonstrate that both temperature and soil moisture, individually and in interaction, significantly influence root metabolic profiles. Our results confirmed (H1) that root metabolic profiles in *R. anthopogon* are strongly influenced by both the individual and interactive effects of temperature and moisture. However, in contrast to H1, temperature was not the dominant driver as we expected. In line with H2, climatic factors interacted to affect a wide range of metabolites across various classes. The changes in both primary and secondary metabolites due to increasing temperature were largely determined by soil moisture availability. In line with H3, soil chemistry also played a role in determining the root metabolome. Contrary to H3, the effects of climate on root metabolomes were not confounded by soil chemistry, emphasizing the dominant role of climatic factors in shaping root metabolic composition in *R. anthopogon*.

### 4.1 Effect of Climatic Factors on Diversity Indices

Root metabolite diversity in *R. anthopogon* shows limited response to temperature, moisture, and their interaction. Metrics like richness, Shannon diversity, and evenness remain stable, likely due to metabolic homeostasis and the buffering capacity of alpine soils (Wu et al. 2022; Wanek et al. 2023). An exception was observed in the beta‐dispersion of metabolites, which decreased with rising temperature under low moisture but increased under high moisture. This suggests that under dry conditions, elevated temperatures may constrain metabolic variability by intensifying abiotic stress, leading to more uniform physiological responses across individuals (Zandalinas et al. 2022). In contrast, under high moisture, warmer temperatures may alleviate cold stress and create more favorable conditions not only for plant metabolic activity but also for biotic interactions, such as herbivory and pathogen pressure (Nataraj et al. 2022). These interactions can induce diverse metabolic responses, thereby increasing variability in metabolite profiles.

Adding soil variables revealed clearer patterns, especially with soil electrical conductivity (EC). Richness declined with soil EC in positive mode. At the same time, Shannon diversity increased and beta‐dispersion decreased in negative mode, highlighting EC's role in nutrient availability and root metabolite regulation, crucial in nutrient‐poor, high‐altitude soils (Wu et al. 2022; Heiniger et al. 2003). Soil EC influences root stoichiometry and, along with temperature and moisture, affects nutrient mobility and metabolism (Ni et al. 2019).

In *R. anthopogon*, root metabolic specialization decreased with higher soil carbon but increased with soil nitrogen, reflecting the stabilizing effect of abundant C and the demand for N‐driven adaptations (Tashi et al. 2016; He et al. 2021). Additionally, functional specialization reduced with rising temperature, especially under low moisture, likely as plants adjust resource allocation strategies in response to warming conditions (Cong et al. 2019).

### 4.2 Effect of Climatic Factors on Root Metabolomic Profiles

Climatic factors reshaped root metabolomic profiles of *R. anthopogon*, with moisture emerging as the strongest driver, followed by its interaction with temperature. This contrasts with studies identifying temperature as the main driver (e.g., Escandón et al. 2018; González‐García et al. 2022), but aligns with other recent work emphasizing moisture's dominant role in plant metabolism (Mdlalose et al. 2022; Nan et al. 2024; Baker et al. 2022). The stronger influence of moisture over temperature on root metabolomes likely reflects the root's primary role in regulating plant water status. Variations in water availability can trigger hydration‐dependent metabolic shifts. Roots exhibit hydropatterning, producing lateral branches along moisture gradients to optimize water uptake (Robbins and Dinneny 2015; Kwasniewski et al. 2016), influencing exudation profiles that attract beneficial microbes (Ali and Glick 2024; Qu et al. 2024). These exudates vary with soil moisture, shaping microbial communities that feedback into plant metabolism (Qu et al. 2024; Wang et al. 2019; Singh et al. 2021). Water stress also leads to osmoprotectant accumulation in both roots and microbes, enhancing resilience (Bhattacharyya et al. 2021; Maitra et al. 2024). Additionally, moisture significantly influences metabolic rates, depending on plant size and stage (Huang et al. 2020).

Notably, strong interactive effects between temperature and moisture suggest that these factors act synergistically to shape root metabolomes, complicating predictions under climate change. Our findings align with emerging evidence that moisture is a dominant driver of root metabolism, while temperature modulates these effects. Understanding these interactions is crucial for forecasting plant responses and developing strategies to support high‐altitude species such as *R. anthopogon*. Such insights can inform conservation, restoration, and climate‐resilient vegetation management in alpine ecosystems.

### 4.3 Soil Chemistry Does Not Mediate the Effects of Climate

The effects of soil chemistry did not override the dominant influence of climate on the root metabolome of *R. anthopogon*, except for soil electrical conductivity (EC), which showed significant effects. Soil EC, an indicator of salinity and nutrient availability, can alter metabolic pathways, especially in nutrient‐limited alpine soils (Anic et al. 2010; Cheng et al. 2018). It modulates stress‐related metabolite production, including flavonoids and phenylpropanoids (Bligny and Aubert 2012; Lu et al. 2023; Magaña Ugarte et al. 2018).

Adjusting for soil chemical variation did not alter climate‐driven metabolomic patterns. This underscores that while edaphic factors contribute to metabolic diversity, climatic variables exert a more direct and systemic influence on root metabolic adaptation in alpine environments. Understanding both drivers is critical for predicting plant responses to environmental change.

### 4.4 Climatic Effects on Individual Metabolites

#### 4.4.1 Climate Affects a High Number of Metabolites From Diverse Chemical Classes

Our findings show that the largest proportion of tentatively annotated primary metabolites affected by climate in *R. anthopogon* includes amino acids, lipids, fatty acids, and organic acids. These compounds are essential for plant adaptation, supporting membrane stability and enhancing stress tolerance (Pollard et al. 2008; Heinemann and Hildebrandt 2021). Amino acids are central to protein synthesis, nitrogen transport and assimilation, and function as signaling molecules in osmotic stress responses (Denby and Last 1999). Fatty acids in lipids serve as carbon reserves in root exudates, enrich soil carbon stocks, and maintain membrane integrity (Couvillion et al. 2024). Organic acids contribute to nutrient mobilization (Jones and Darrah 1994), stress mitigation (Hadavi and Ghazijahani 2022; Wu et al. 2018), and regulate energy balance, membrane fluidity, and protein synthesis (Williamson et al. 2002). As temperature and moisture vary, plants adjust these metabolic pathways, leading to changes in metabolite concentrations.

Climate also significantly shaped secondary metabolites, including flavonoids, glycosides, terpenoids, phenolics, and coumarins, key players in root function and plant‐environment interactions. Flavonoids modulate auxin transport, symbiotic signaling, and oxidative stress responses (Abdel‐Lateif et al. 2012; Weston and Mathesius 2013; Hassan and Mathesius 2012). Glycosides facilitate metabolite transport and symbiosis, with climate‐driven variation indicating their role in long‐term adaptation (Yoshitama 2000; Li et al. 2021). Terpenoid synthesis is governed by carbon and nitrogen availability, linking defense responses to nutrient dynamics (Naji et al. 2024; Yazaki et al. 2017; Sun et al. 2023). Phenolics serve as antioxidants, regulate hormone signaling, and influence microbial community dynamics (Yoshitama 2000; Sato et al. 2014; Laftouhi, Eloutassi, Drioua, et al. 2023; Laftouhi, Eloutassi, Ech‐Chihbi, et al. 2023). Coumarins aid iron acquisition and microbial balance under changing environmental conditions (Stringlis et al. 2019; Ihnatowicz et al. 2024; Sisó‐Terraza et al. 2016).

In line with H2, we do find an increase in the production of glycosides and flavonoids with increasing temperature and moisture. Together, these shifts reflect an integrated metabolic strategy by *R. anthopogon* to withstand climatic stress, highlighting the importance of both primary and secondary metabolite plasticity in high‐altitude plant adaptation.

#### 4.4.2 Effect of Climate on Metabolic Pathways and Metabolites Involved in Them

Pathway analysis revealed that metabolites affected by climate in *R. anthopogon* mapped to 23 pathways. Among these, the metabolism of branched‐chain amino acids (BCAA; valine, leucine, and isoleucine) was prominently impacted. These amino acids are vital under stress, especially when carbohydrate supply is limited (Schertl et al. 2017; Latimer et al. 2018) and are essential for root function and amino acid homeostasis (Schuster and Binder 2005). In our study, leucine and isoleucine levels increased with increasing temperature and moisture, suggesting metabolic activation during heat stress (Liu et al. 2024; Planchet and Limami 2015). They also act as alternative energy sources and stress signaling molecules (Heinemann and Hildebrandt 2021; Samsami and Maali‐Amiri 2024; Joshi et al. 2021; Joller et al. 2024; Kolupaev et al. 2024).

Other responsive pathways included monoterpenoid, glycerophospholipid, phenylpropanoid, and flavonoid biosynthesis. Beta‐myrcene, a monoterpene, increased under high temperature and low moisture, aiding stress tolerance via enhanced photosynthesis and antioxidant defense (Bertamini et al. 2019; Dobhal et al. 2024). Flavonoid biosynthesis, especially of myricetin and taxifolin, also increased under high temperature and low moisture, offering protection from oxidative and UV stress (Gouot et al. 2020; Ramachandran and Raj 2024; Patil et al. 2024). Phosphatidylethanolamine (PE), linked to glycerophospholipid metabolism, also rose under these conditions, indicating membrane stabilization (Dornbos et al. 1989).

In contrast, phenylpropanoid biosynthesis (e.g., ferulic acid and syringin) declined under higher temperatures and lower moisture, indicating vulnerability to climatic shifts (Gashu et al. 2023). Vitamin metabolism was also sensitive. Pyridoxine (vitamin B6) and pantothenic acid declined under heat and moisture stress, indicating potential loss of enzymatic efficiency (McCormick and Chen 1999; Dell'Aglio et al. 2017; Chakauya et al. 2008; Chakauya et al. 2006). Meanwhile, asparagine and ornithine increased with temperature and low moisture. Asparagine facilitates nitrogen transport (Gaufichon et al. 2016; Han et al. 2022), and ornithine supports polyamine biosynthesis and stress response (Majumdar et al. 2015, 2016). Their rise reflects nitrogen recycling and osmotic regulation under stress (Martínez‐Lorente et al. 2024; Hildebrandt et al. 2015; Secomandi et al. 2025).

Overall, climate‐induced shifts in BCAA, flavonoid, monoterpenoid, and lipid pathways, along with declines in vitamins, underscore *R. anthopogon's* metabolic flexibility. These adjustments support stress resilience and provide mechanistic insight for improving tolerance in other high‐altitude climate‐sensitive species.

#### 4.4.3 Effect of Climate on Metabolites Not Represented in Pathway Analysis

Our study demonstrates that elevated temperatures and reduced moisture levels significantly reshape the root metabolome in *R. anthopogon*, particularly affecting amino acids, fatty acids, and secondary metabolites.

Alpha amino acids increased under high temperature and high moisture, reflecting enhanced protein biosynthesis, stress adaptation, and energy compensation via alternative respiratory substrates such as proline and GABA (Gamma Amino Butyric Acid) (Planchet and Limami 2015). These amino acids also function as signaling molecules, modulating pathways that govern energy balance and stress responses (Heinemann and Hildebrandt 2021; Yuxiao et al. 2023).

Fatty acids also serve as precursors for stress‐related signaling molecules (Liang et al. 2023; Hou et al. 2016). Elevated temperatures promoted unsaturated fatty acid synthesis via the eukaryotic pathway to maintain membrane fluidity (Li et al. 2015, 2016).

Arginine and its derivatives showed a marked increase under high temperature and water stress, aligning with their roles in nitrogen storage, signaling, and abiotic stress resilience via polyamine and nitric oxide synthesis (Winter et al. 2015; de Sousa Araújo et al. 2019). Arginine metabolism is pivotal in plant stress responses, particularly through the production of polyamines and nitric oxide, which are derived from arginine and play roles in stress signaling and adaptation (Shi and Chan 2013).

Lipid metabolism also responded strongly to climate conditions. Saccharolipids decreased under high temperature and low moisture, while fatty acids increased. These lipids are essential for maintaining membrane integrity and energy storage, especially under thermal and drought stress (Bhattacharya 2022; Kim 2020; Schmid and Ohlrogge 2002). Saccharolipids play a significant role in plant root metabolism, particularly in the context of nutrient uptake (Pandey et al. 2025), stress response (Pandey et al. 2025; Liu et al. 2021), metabolic regulation (Esterhuizen et al. 2024), and interactions with the rhizosphere (Hennion et al. 2019; Read et al. 2003).

In *R. anthopogon*, secondary metabolites such as triterpenoids, flavonols, coumarin glycosides, and phenolic glycosides were differentially affected by climate. Flavanols, phenolic, and coumarin glycosides decreased with high temperature and high moisture, whereas triterpenoid increased with rising temperature and high moisture. These compounds play key roles in defense, signaling, and stress tolerance (Ghadirnezhad Shiade et al. 2023; Mukherjee et al. 2016). Flavonols support photosynthesis and cold tolerance (Kitashova et al. 2024), while coumarins act as antioxidants and regulators of stress response (Alamgir 2018). Through the mevalonate pathway, triterpenoids enhance structural and pathogen resistance (Jozwiak et al. 2020). Phenolic glycosides assist in oxidative stress defense and environmental adaptation (Mohiuddin 2019). Their accumulation is favored under moderate to high temperatures (~15°C) and drought (Sun et al. 2022; Rani et al. 2017). However, enhanced secondary metabolism may compete with growth and reproduction, potentially impacting overall plant fitness and ecological interactions (Srivastava et al. 2021; Singh and Choudhary 2023).

In high‐altitude species like *R. anthopogon*, these climate‐driven metabolic adjustments are crucial for survival. Increased production of flavonols, coumarins, and phenolics likely supports antioxidant defenses, photoprotection, and osmotic balance under intense UV and limited water availability. Lipid remodeling further aids membrane stability under freezing conditions. Understanding these adaptations offers valuable insights for improving the resilience of alpine and other climate‐sensitive plants.

#### 4.4.4 Correlation Network Analysis of Metabolites

The correlation network analysis across both ionization modes revealed a highly interconnected metabolome, with no distinct clustering among chemical classes, suggesting that plant metabolic pathways operate in an integrated manner. Hub metabolites—alkaloids, amino acids, flavonoids, phenolics, and glycosides—exhibited high connectivity and functioned as regulatory nodes coordinating diverse physiological processes.

Tropine, an alkaloid, plays a role in the biosynthesis of pharmacologically significant tropane alkaloids such as hyoscyamine and scopolamine (Hu et al. 2023; Jamali et al. 2015). Alkaloid levels rose under high temperature and low moisture, aligning with stress‐induced accumulation observed in a study by Alhaithloul et al. (2019). These compounds are also affected by microbial associations and contribute to plant stress and defense responses (Li et al. 2022).

The amino acid trihydroxy‐1‐oxa‐hexazacyclotritriacontane‐hexone also increased under combined heat and drought stress. Amino acids serve as precursors and signaling molecules with strong genetic and metabolic links, especially during stress conditions (Batushansky et al. 2015; Joller et al. 2024). They also act as osmoprotectants in some species (Yin et al. 2024; Alhaithloul et al. 2019).

In contrast, levels of phenolics, flavonoids, and glycosides declined under high temperature and low moisture. Resveratroloside, a phenolic compound synthesized in response to stress, typically increases under such conditions (Stuart and Robb 2013; Wenzel and Somoza 2005; Chalker‐Scott and Fuchigami 2018), yet its reduction suggests complex interactions between heat and moisture. Phenolics also mediate plant–microbe interactions and defense responses (Lone et al. 2024; Tuladhar et al. 2021), and their decline contradicts their usual role during stress (Mansinhos et al. 2022; Qaderi et al. 2023).

Flavonoids, which play a role in hormone signaling and plant‐microbe interactions (Yonekura‐Sakakibara et al. 2008; Wang et al. 2022), also decreased. Although high temperatures can stimulate their production, combined drought appears to suppress it (Laftouhi, Eloutassi, Drioua, et al. 2023; Laftouhi, Eloutassi, Ech‐Chihbi, et al. 2023; Alhaithloul et al. 2019). Similarly, glycosides, which contribute to solubility, stability, nitrogen recycling, and stress tolerance (Le Roy et al. 2016; Wei et al. 2021; Pičmanová et al. 2015), were reduced, likely due to altered precursor availability and disrupted regulatory pathways (Singh and Choudhary 2023).

In *R. anthopogon* roots, we found that the different metabolite classes were interrelated, and there were no stronger relationships between specific metabolite classes, suggesting a highly integrated metabolic network. Metabolites from different classes likely interact and influence each other, reflecting the complexity and interconnectedness of metabolic pathways. This result suggests that the regulation of metabolites is systemic rather than isolated to specific classes. It implies that metabolic processes are coordinated across different pathways, potentially through shared regulatory mechanisms or common metabolic intermediates. To fully understand the metabolic dynamics, an approach that considers the entire metabolic network rather than focusing on individual classes is essential. This can help identify key regulatory nodes and enhance understanding of the overall metabolomic dynamics. Therefore, we conclude that hub metabolites may serve as ideal predictors of plant responses to climate change. However, further research is needed to characterize them and define their roles precisely in the context of root metabolomics. Overall, these findings underscore the integrated nature of plant metabolite classes and illustrate how environmental stress influences them through complex, interconnected regulatory mechanisms.

### 4.5 Implications of Root Metabolomic Insights

The metabolomic insights from this study can have valuable applications across various fields. In ecological restoration, understanding how root metabolic profiles respond to natural variations in temperature, moisture, and soil chemistry can help select stress‐adapted genotypes for revegetation in degraded alpine and subalpine environments (Lin et al. 2022; Du et al. 2024). Specifically, identifying metabolite signatures linked to drought resilience and nutrient availability can guide the selection of populations and restoration strategies that support long‐term ecosystem stability. In pharmaceutical prospecting, the discovery of climate‐sensitive secondary metabolites such as flavonoids, terpenoids, alkaloids, and glycosides highlights *R. anthopogon* as a potential source of bioactive compounds with antioxidant, antimicrobial, or anti‐inflammatory properties (Innocenti et al. 2010; Baral et al. 2015). Since environmental stress often boosts secondary metabolite production, insights from this study can help develop targeted harvesting or cultivation methods to maximize therapeutic compound yields (Pant et al. 2021; Yeshi et al. 2022). Ultimately, these findings provide a molecular framework for assessing plant health and predicting responses to changing precipitation and temperature patterns in climate‐resilient shrubland management (Sardans et al. 2020). The identification of key metabolic pathways and hub compounds offers potential biomarkers for monitoring plant stress and adaptation, guiding management practices such as grazing regulation, assisted migration, or soil amendment to sustain functional shrubland ecosystems under climate pressures (Samsami and Maali‐Amiri 2024).

## 5 Conclusion

This research provides insights into how climate variables, particularly temperature and soil moisture, impact the root metabolomic profiles of *R. anthopogon*, a high‐altitude plant. Our findings show that climatic factors significantly affect metabolite compositions in plant roots, with soil moisture emerging as the key driver of metabolic changes, often intensified by temperature fluctuations.

Water availability is crucial in high‐altitude ecosystems since moisture‐dependent metabolic pathways related to amino acids and microbial interactions are vulnerable. The interplay between moisture and temperature underscores the complexity of environmental stress responses, emphasizing the need to analyze these factors together.

Temperature affects root metabolism through thermosensitive pathways, driving increased production of stress‐responsive metabolites like branched‐chain amino acids (BCAAs), unsaturated fatty acids, and alkaloids. These are vital for membrane fluidity and energy production under photosynthetic stress.

Secondary metabolites such as flavonoids, coumarins, and phenolic glycosides respond differently to specific temperature and moisture combinations. They play key roles in antioxidant defense and UV protection, which are essential for survival at high elevations.

Also, soil chemistry did not influence root metabolomes (except for soil EC), and climatic factors exert a more profound and immediate impact. Correlation network analysis identified key metabolites like alkaloids, amino acids, and flavonoids, indicating cohesive functioning within plant metabolic networks.

In summary, root metabolic responses in high‐altitude plants are highly sensitive to climatic changes. Understanding these metabolomic responses is vital for anticipating plant adaptation strategies as climate change continues to affect mountainous environments, thus guiding conservation and agricultural resilience efforts.

## Author Contributions

S.B., D.T., and Z.M. conceptualized the study; field sampling was conducted by S.B., N.R., and D.T.; N.R. and S.B. prepared the samples for UPLC analysis, while N.R., D.T., and J.S. optimized the UPLC‐MS method for analysis; UPLC‐MS analysis was performed by J.S. and T.C.; the raw data were processed to obtain metabolite feature data by S.B., D.T., and J.S.; data analysis was carried out by S.B. with the help of D.T. and Z.M.; S.B. led the writing, with in‐depth editing and suggestions from D.T. and Z.M.; D.T. and Z.M. are the senior authors; all authors approved the submission.

## Conflicts of Interest

The authors declare no conflicts of interest.

## Supporting information


**Data S1:**Supporting Information.

## Data Availability

The datasets created and analyzed in this study are currently available in the Zenodo repository. (doi: 10.5281/zenodo.16568398).
