# Supplementary material for: Soil Moisture and Its Interaction With Temperature Determine Root Metabolomes of a Himalayan Alpine Shrub
Source: Physiol Plant. 2025 Aug 12;177(4):e70444. doi: 10.1111/ppl.70444 (PMC12344346; doi:10.1111/ppl.70444)
Supplement: Supplementary file 1 — Data S1:Supporting Information. [file PPL-177-e70444-s001.pdf]

SUPPLEMENTARY INFORMATION

Soil moisture and its interaction with temperature determine root metabolomes of a Himalayan alpine shrub

Sayantika Banerjee[1,2*], Nikita Rathore[2], Jaroslav Semerád[3], Tomáš Cajthaml[3], Zuzana Münzbergová[1,2]†, Dinesh Thakur[2]†

1. Department of Botany, Faculty of Science, Charles University in Prague, Benátská 2, 128 01 Praha 2, Czech Republic.
2. Institute of Botany, Czech Academy of Sciences, v. v. i., Zámek 1, 252 43 Průhonice, Czech Republic.
3. Microbiology of the Czech Academy of Sciences, Vídeňská 1083, 142 20 Prague, Czech Republic.

† Equal senior authorship

Figure S1: Correlation matrix results of climate variables

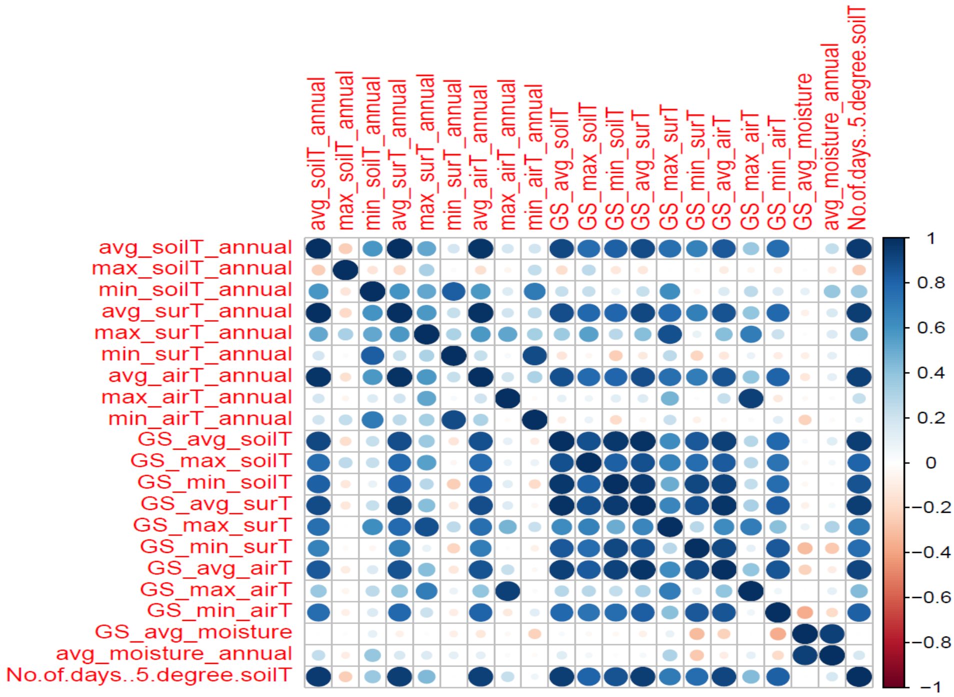

Figure S2: Direction of effects of climate and soil variables on diversity indices of root metabolites

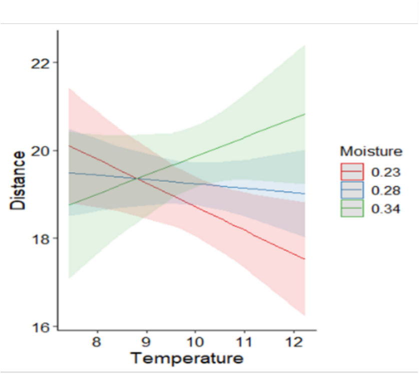

2a: Direction of climatic effect on beta dispersion_positive ionization mode

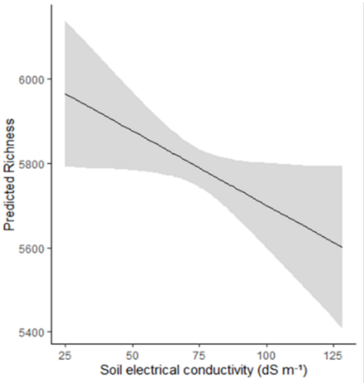

2b: Direction of Soil EC on richness_positive ionization mode

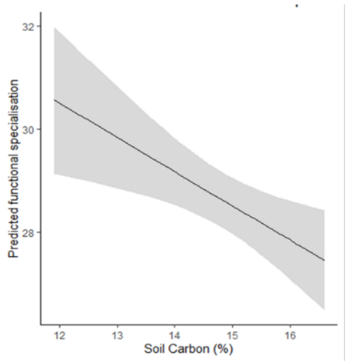

2c: Direction of Soil C on specialisation_positive ionization mode

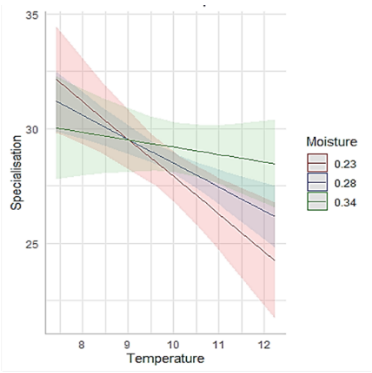

2d: Direction of temperature effect on specialisation_positive ionization mode

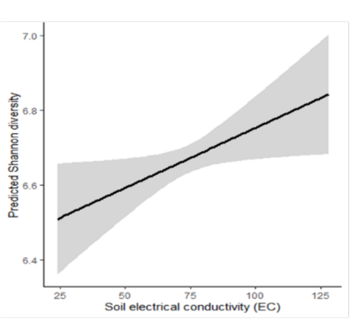

2e: Direction of Soil EC effect on Shannon diversity_negative ionization mode

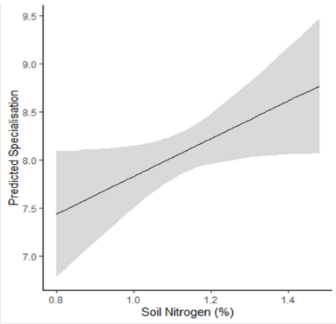

2f: Direction of Soil N effect on specialisation_negative ionization mode

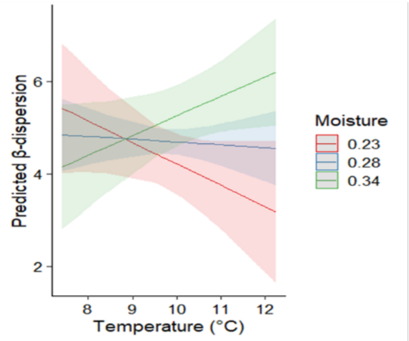

2g: Direction of climate effect on beta dispersion_negative ionization mode

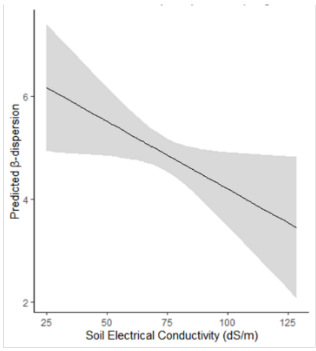

2h: Direction of Soil EC on beta dispersion_negative ionization mode

Figure S3: Venn diagram showing number of metabolites significantly affected by temperature, moisture and their interaction (temperature × moisture). The overlapping areas represent metabolites affected by two or all three of the predictors.

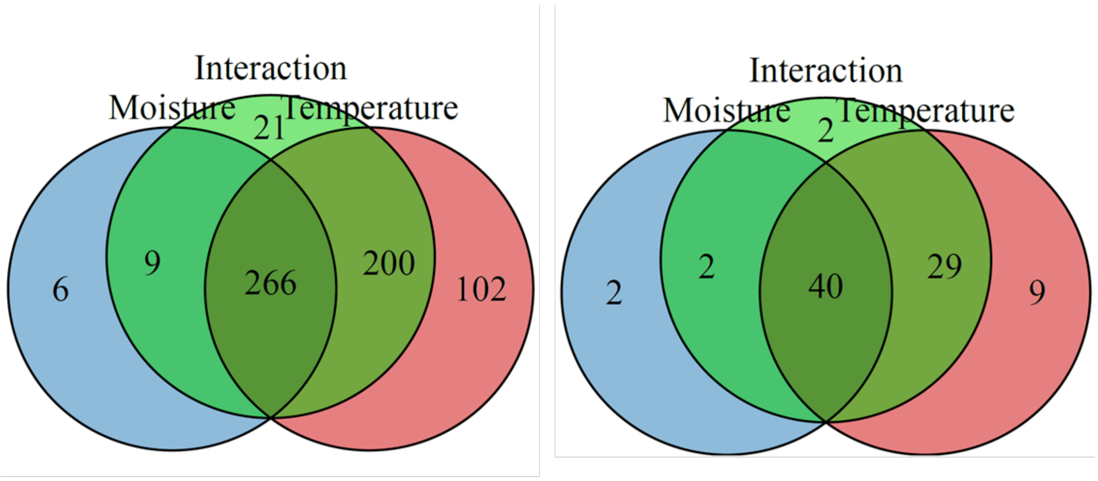

a: Positive Ionisation **b: Negative Ionisation**

Figure S4: Bubble plot representing metabolomic pathways, where the x-axis represents pathway impact, indicating the extent to which each pathway is influenced or altered. The y-axis displays -log10(p), reflecting the statistical significance of each pathway, with higher values corresponding to stronger significance (lower p-values). The color of each bubble ranges from yellow to red, with red indicating highly significant pathways and yellow representing less significant ones. Bubble size denotes the strength of the pathway. Number inside each bubble corresponds to the name of the pathway in table given below (Table 6).

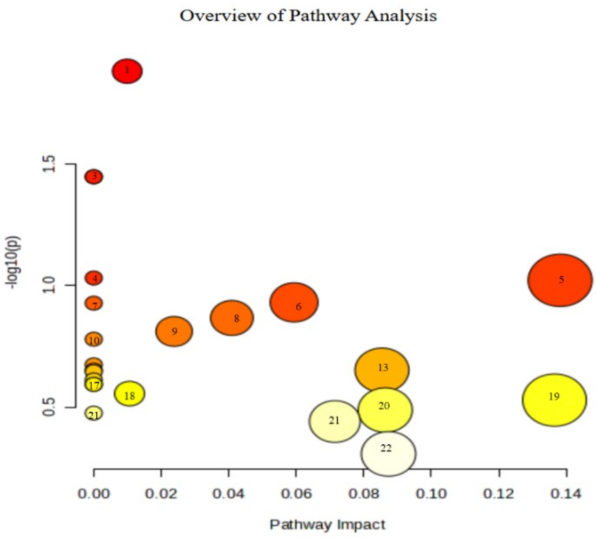

Figure S5: Patterns of changes in response to temperature, moisture and their interaction in some of the metabolites present in the metabolomic pathway analysis. The plots are based on the significant effects detected in the linear mixed effects models.

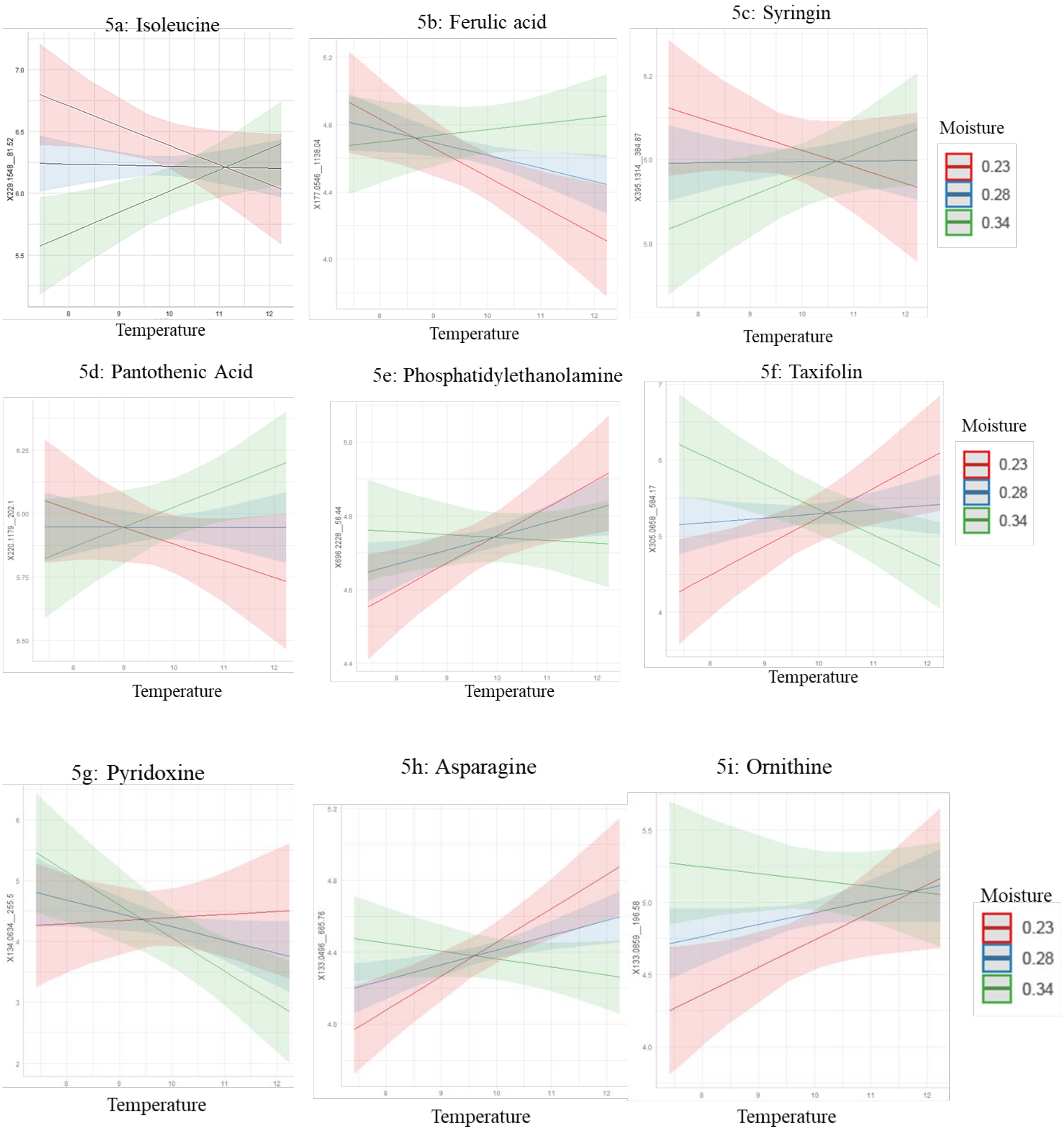

Figure S6: Patterns of changes in response to temperature, moisture and interaction in some of the detected and annotated metabolites. The plots are based on the significant effects detected in the linear mixed effects models.

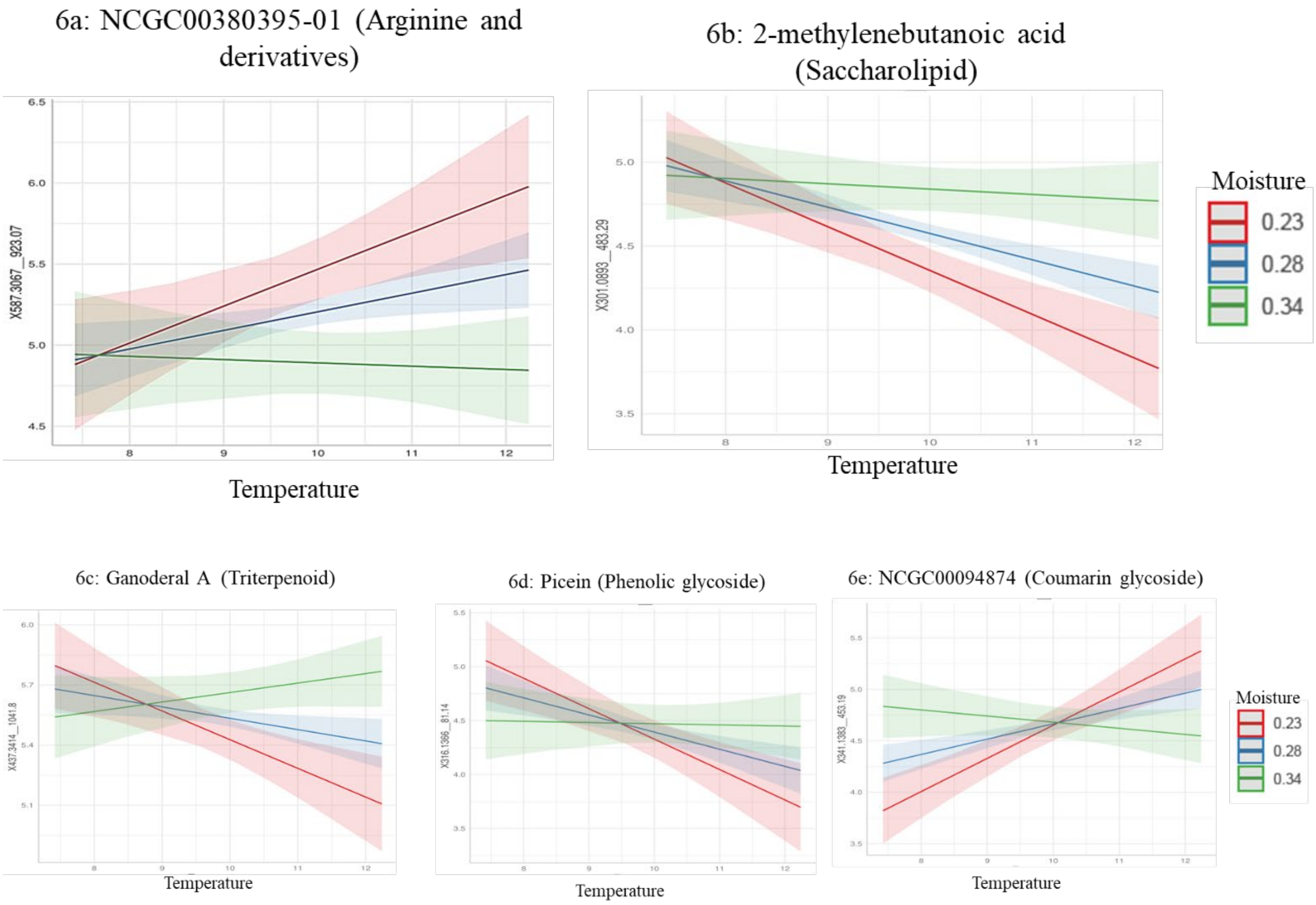

Figure S7: The correlation network of metabolites in positive ionization mode (a) and negative ionization mode (b) illustrates the relationships among detected metabolites, which are classified into major chemical groups. In these networks, nodes represent metabolites and are sized according to their connectivity (degree centrality). Edges indicate significant correlations, with green edges representing positive correlations and red edges representing negative correlations, scaled by interaction strength. To enhance visibility, node sizes are adjusted in each mode plot to account for the differing number of metabolites.

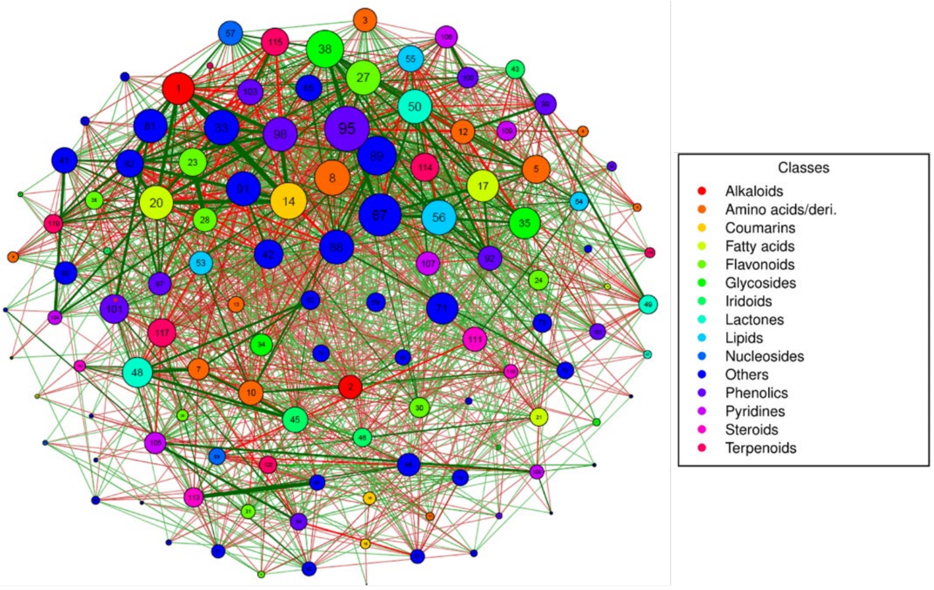

7a: Positive Ionisation

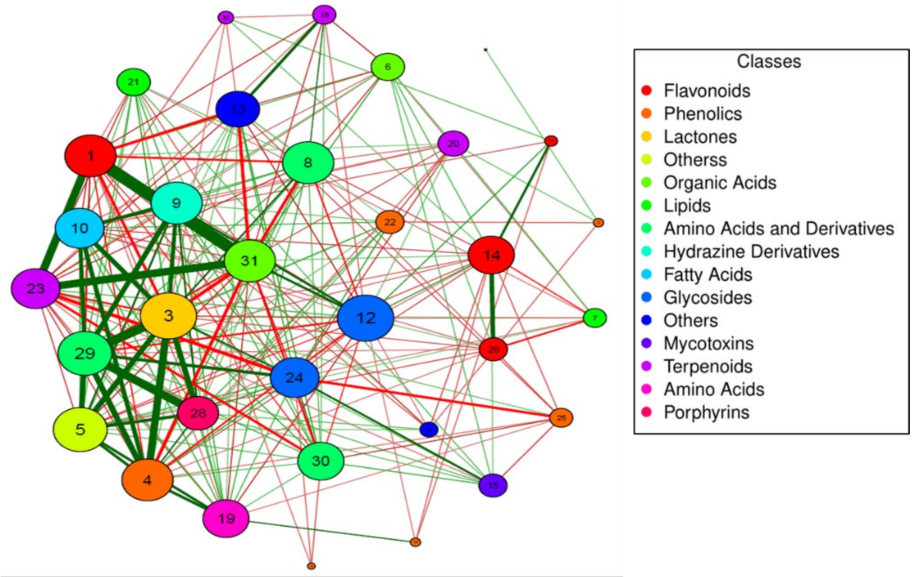

7b: Negative Ionisation

Table S1: Number of metabolites annotated from each chemical affected by climate in positive ionisation mode dataset.

Metabolite Class	Temp.	Moist.	Temp × Moist
Flavonoids	22	18	18
Amino acids and derivatives	10	10	10
Amino acid derivatives	8	6	6
Coumarins	6	6	4
Phenylpropanoids	6	5	5
Furans	5	3	3
Terpenoids	4	4	5
Monoterpenoids	4	3	4
Phenylketones	2	1	3
Lipids	3	2	3
Benzopyrans	1	2	1
Sesquiterpenoids	2	2	2
Aldehydes	-	2	1
Steroids	-	1	2
Cyclohexenones and derivatives	2	2	2
Peptides	2	2	2
Azines	1	1	1
Pyridines and derivatives	1	1	1
Purines and derivatives	1	1	1
Benzazoles	1	1	1
Quinoline alkaloids	1	1	1
Phenolic compounds	1	1	1

Table S2: Number of metabolites annotated from each chemical affected by climate in negative ionisation mode dataset.

Metabolite Class	Temp.	Moist.	Temp × Moist
Diols	1	1	2
Phospholipids	1	1	2
Hydroxy Fatty Acids	0	0	2
Amino Acids	2	0	3
Tetrapyrroles	1	1	2
Lactones	2	0	3
Cyclic Peptides	3	0	1
Lipids	3	1	1
Phenolic Acids	2	0	0
Flavonoids	2	2	5
Not Classified	2	1	4
Phenolics	2	1	2
Isoflavonoids	4	1	1

Macrolides	3	0	1
Fatty Acids	2	1	1
Glycosides	5	2	2
Naphthalenes	1	1	2
Organic Acids	2	1	1
Phenyl Derivatives	2	1	1
Terpenoids	4	2	4
